# Laparoscopic colectomy after transcatheter aortic valve implantation in an elderly patient with obstructive descending colon cancer and severe aortic stenosis: a case report

**DOI:** 10.1186/s40792-019-0662-1

**Published:** 2019-06-24

**Authors:** Koki Tamai, Shu Okamura, Tomohiro Kitahara, Takayuki Minoji, Hiroyuki Takabatake, Noriyuki Watanabe, Noriyuki Yamamura, Nariaki Fukuchi, Chikara Ebisui, Hideoki Yokouchi, Masaki Tsuda, Isamu Mizote, Masakatsu Kinuta

**Affiliations:** 1 Department of Surgery, Suita Municipal Hospital, Kishibeshinmachi 5-7, Suita, Osaka 564-8567 Japan; 2 Department of Cardiovascular Medicine, Osaka University Graduate School of Medicine, Yamadaoka 2-2, Suita, Osaka 565-0871 Japan

**Keywords:** Laparoscopic colectomy, Severe aortic stenosis, Transcatheter aortic valve implantation

## Abstract

**Background:**

The demand for laparoscopic colectomy is increasing due to greater number of elderly colon cancer patients, and it is important to evaluate existing comorbidities to ensure perioperative safety. Aortic stenosis (AS) is one of the most common heart diseases in the elderly, and elderly cancer patients with severe AS may be considered ineligible for optimal cancer treatment if they cannot endure surgical aortic valve replacement (SAVR). Recently, transcatheter aortic valve implantation (TAVI) has become a valid option in patients who are high risk for SAVR. We herein present the first case of an elderly cancer patient with severe AS who underwent laparoscopic colectomy after TAVI.

**Case presentation:**

An 87-year-old woman with a history of multiple cardiovascular diseases was diagnosed with obstructive descending colon cancer and initially underwent colonic stenting. However, as preoperative echocardiography revealed severe AS, she underwent TAVI prior to the colectomy to reduce perioperative risk. TAVI was chosen instead of SAVR due to high SAVR mortality risk, and laparoscopic colectomy was performed 22 days after TAVI. Her postoperative course was uneventful, and she was discharged 14 days later without any deterioration in general condition. No recurrence was observed at more than 1 year, even without adjuvant therapy.

**Conclusion:**

TAVI facilitated subsequent laparoscopic colectomy in an elderly cancer patient with severe AS. Our case report shows that TAVI may enable further cancer treatment even in patients with severe AS, who may otherwise be considered not suitable for such treatments.

## Background

Increasing life expectancy has led to a greater number of elderly cancer patients requiring colorectal surgery, and laparoscopic procedures are preferred due to the need for minimally invasive surgery in the elderly. Additionally, careful attention should be paid to the presence of comorbid cardiopulmonary disease because a pneumoperitoneum decreases both cardiac output and pulmonary compliance.

Aortic stenosis (AS) is estimated to be present in 4–6% of the elderly. Although surgical aortic valve replacement (SAVR) has been the standard treatment in severe AS patients, the patients with advanced age, heart failure, or cancer are sometimes considered ineligible for the procedure due to its high perioperative mortality and morbidity risk. Thus, cancer patients ineligible for SAVR may not be able to undergo sufficient cancer treatment.

Recently, transcatheter aortic valve implantation (TAVI) has become a valid option in patients who are high risk for SAVR. While TAVI has facilitated sufficient and subsequent cancer treatment, little is known on the usefulness of TAVI prior to cancer surgery. Herein, we describe the case of an elderly cancer patient with severe AS who underwent laparoscopic colectomy after TAVI.

## Case presentation

**Fig. 1:**
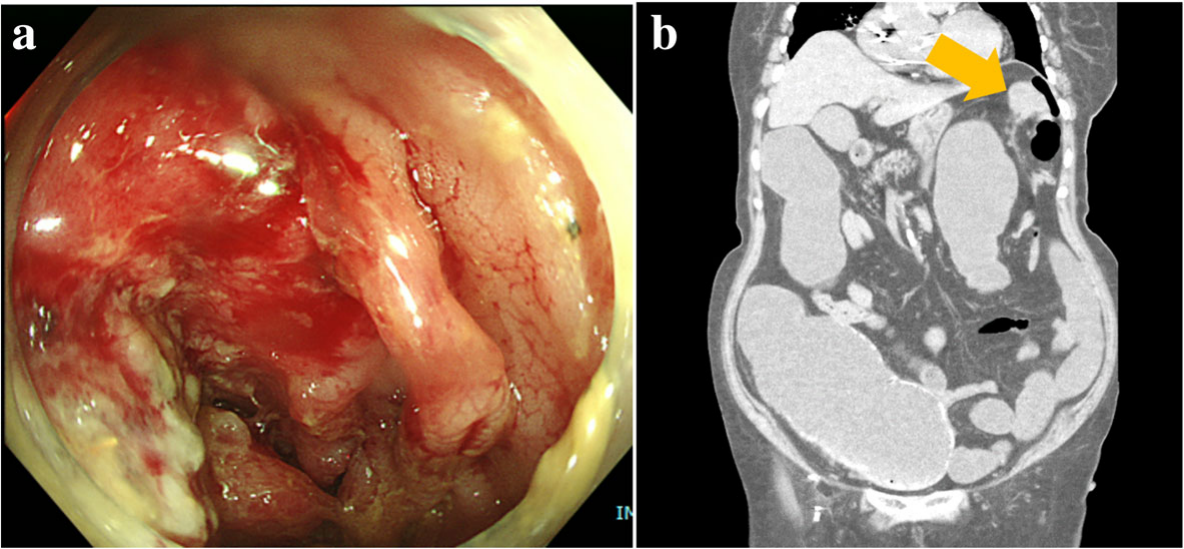
Preoperative colonoscopy and computed tomography findings. **a** Colonoscopy confirmed descending colon cancer and obstruction. **b** Computed tomography revealed an obstructive ileus due to descending colon cancer (yellow arrow)

**Fig. 2:**
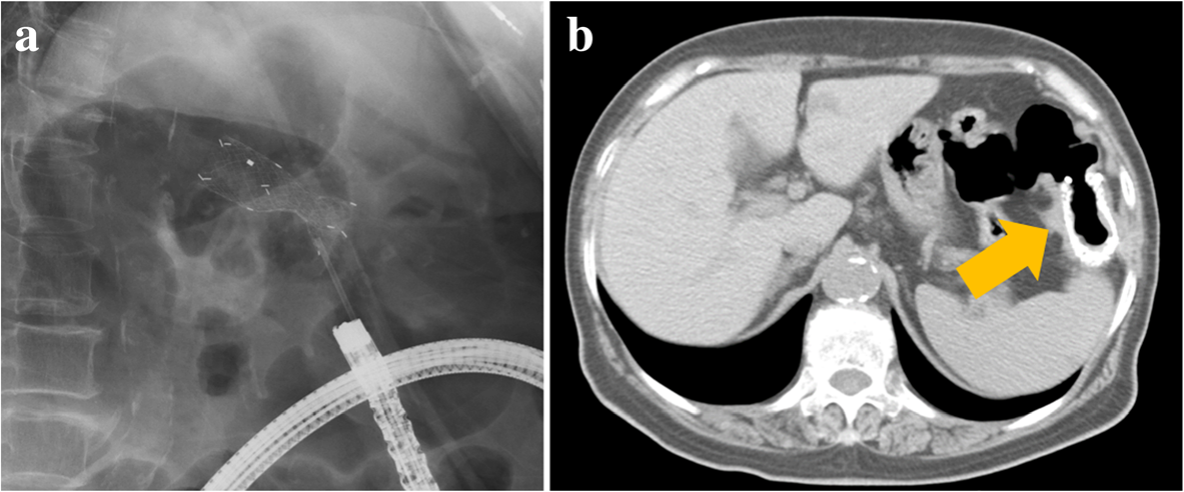
Fluoroscopy and computed tomography findings after colonic stent placement. **a** Colonic stent was placed in the descending colon under fluoroscopic guidance. **b** Colonic stent (yellow arrow) reduced the obstruction

An 87-year-old woman was referred to our hospital in poor general condition (Eastern Cooperative Oncology Group performance status 3) with dyschezia and loss of appetite for the past week. A colonoscopy confirmed an obstructive descending colon cancer that could not be penetrated by the colonoscope (Fig. 1a), and computed tomography revealed an obstructive ileus (Fig. 1b). The patient’s medical history included coronary artery bypass surgery 11 years ago, surgery for dissecting aortic aneurysm 8 years ago, pacemaker implantation for sick sinus syndrome a year ago, chronic renal failure, and type 2 diabetes. Although a colectomy to reduce the obstruction was required at the earliest possible time, we first placed a colonic stent on the first day of admission to gain some time for preoperative evaluation to reduce perioperative risk (Fig. 2a, b). Then, preoperative echocardiography on the second day after admission revealed severe AS with an aortic valve area of 0.78 cm^2^, mean aortic pressure gradient of 48 mmHg, a maximum jet velocity of 4.2 m/s, and an ejection fraction of 63%. Although manifestations of severe AS were not clear due to her reduced activity levels, we thought that treating severe AS prior to the colectomy was not entirely wrong because having symptoms were undeniable, and the brain natriuretic peptide, which is one of the risk factors for complication of AS, was high (216.1 pg/mL). Furthermore, the high surgical risk for colectomy calculated from the American College of Surgeons National Surgical Improvement Program Surgical risk calculator (20.9% of serious complication and 5.0% of mortality risk) was also a reason to prioritize the treatment of AS. It is because the deterioration of the general condition, which was one of the main factors of surgical risk, might be caused by severe AS. However, as SAVR mortality risk was evaluated to be high at 8.7% (according to the European system for cardiac operative risk evaluation II) and adverse events and surgical stress after SAVR may delay or prevent the colectomy, we proceeded with TAVI instead of SAVR.

**Fig. 3:**
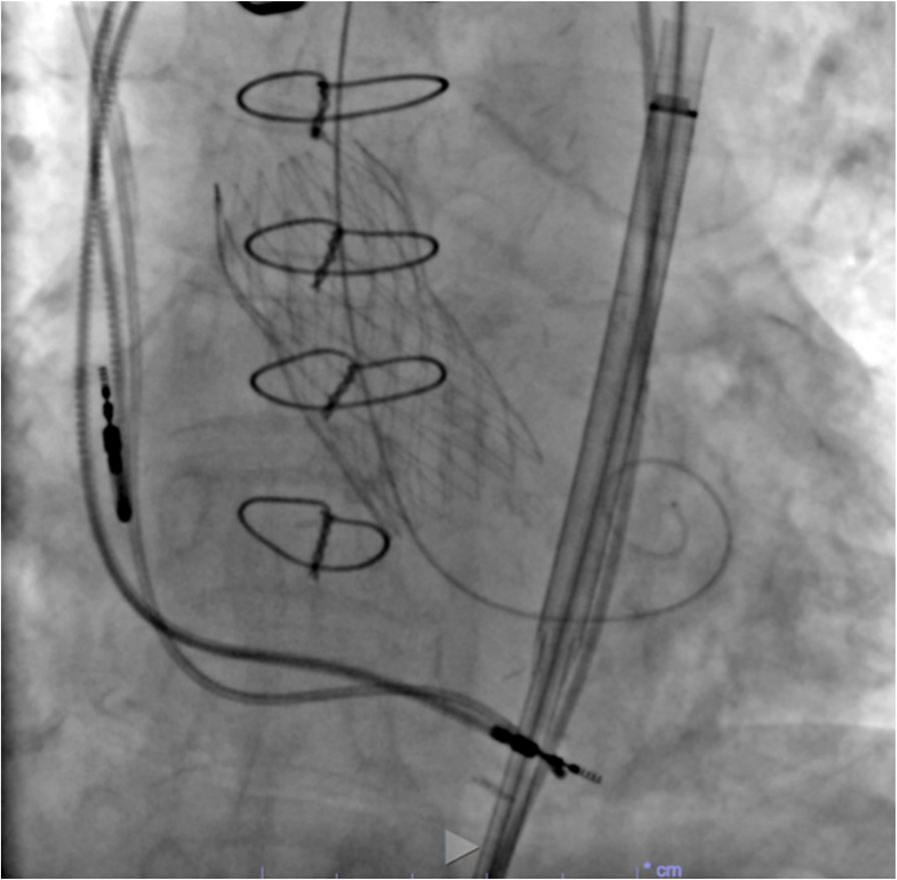
Fluoroscopy during transcatheter aortic valve implantation with a self-expanding device (26-mm Evolut R valve)

The patient underwent TAVI through the right femoral artery on day 31 after colonic stenting. A 26-mm Evolut R valve was placed under fluoroscopic guidance (Fig. 3) and echocardiography 7 days after the procedure showed an improvement in the mean aortic pressure gradient to 7 mmHg with an aortic valve area of 1.82 cm^2^ and the ejection fraction to 70%.

To reduce probable tumor bleeding, we administered aspirin alone from TAVI until colectomy, even though dual antiplatelet therapy is recommended. The patient received 10,000 unit/day heparin administration for 7 days instead of aspirin until 5 h prior to surgery and then underwent laparoscopic colectomy 22 days after TAVI. We ligated left colic artery, which was the main feeding artery of the tumor, and performed D3 lymph node dissection using a 5-port technique in the supine position. The colon was resected with sufficient distal and proximal margins from the tumor and anastomosed. The operation time was 346 min and the estimated blood loss was 10 ml. Pathologically, the tumor was diagnosed as T4a, N1b (3/32), M0, Stage IIIB. Her postoperative course was unremarkable, and she was discharged 14 days after colectomy. No recurrence was observed for more than 1 year without adjuvant therapy.

## Discussion

Laparoscopic surgery for colorectal cancer is a minimally invasive procedure that is associated with less postoperative pain, fewer complications, faster recovery, and greater early social rehabilitation [1, 2]. Therefore, it is suitable for high-risk patients, especially those with frailty and in the elderly. In recent years, laparoscopic surgery in such high-risk populations has been reported to have fewer complications and lower mortality rates compared with open laparotomy [3–5]. Meanwhile, in patients with low cardiac or pulmonary function, a pneumoperitoneum would exacerbate cardiopulmonary function by decreasing cardiac output and pulmonary compliance [6]. Therefore, it is essential to evaluate cardiopulmonary function prior to laparoscopic surgery. Our patient had a history of multiple cardiovascular diseases, and we performed echocardiography to evaluate her current cardiovascular status, which incidentally revealed severe AS. Symptoms of AS such as shortness of breath and palpitation at the time of exertion may not be readily apparent in the elderly with reduced activity, and in cancer patients, anemia and fatigue caused by the disease itself can mask the presence of cardiopulmonary symptoms. Our case serves to confirm the importance of careful preoperative evaluation, especially in elderly cancer patients.

AS is a narrowing of the aortic valve opening that leads to the obstruction of the left ventricular outflow, subsequent left ventricular systolic dysfunction, and ultimately heart failure in the long-term. In patients undergoing non-cardiac surgery, severe AS is a known high-risk factor of mortality and morbidity because it reduces coronary perfusion under surgical stress and hemodynamic changes. The European Society of Cardiology guidelines recommend SAVR in symptomatic severe AS patients prior to non-cardiac surgery [7], while in asymptomatic severe AS patients, SAVR is recommended only when the risk of non-cardiac surgery is high, and the risk of SAVR is low. Our patient did not meet the absolute indications for SAVR because of her poor symptoms; however, we thought it was better to prioritize the treatment of AS using TAVI over colectomy because AS severity and symptoms, which can increase the risk of colectomy, may not be apparent in such an elderly cancer patient.

TAVI is a minimally invasive approach for replacing the aortic valve that differs from SAVR in that it requires no cardiac arrest, extracorporeal circulation, or thoracotomy. In high-risk or ineligible patients, the PARTNER trial showed that treatment outcomes, including mortality rates, LV function recovery, and morbidity after TAVI were either comparable to or superior to those of conventional therapy [8–10]. Therefore, TAVI has become the standard alternative treatment in high-risk surgical patients. Furthermore, specific advantages of TAVI have been reported in cancer patients, as it does not require cardiopulmonary bypass (CPB). These include a possible reduction in the risks associated with tumor bleeding due to anticoagulant disorders or anticoagulants administration, and tumor dissemination due to immunosuppressive and inflammatory effects of CPB [11, 12]. In patients with severe AS requiring cancer treatment, TAVI not only enables a smooth transition to the next treatment strategy due to its minimally invasive nature but also confers an oncological advantage. Thus, TAVI was considered most suitable for our patient. Importantly, our patient felt an improvement in her physical condition after TAVI even though there were no obvious symptoms, and our case serves to highlight the fact that, in addition to careful preoperative evaluation, it is also necessary to make prudent decisions on treatment strategy in elderly patients whose symptoms are often less apparent.

We prescribed a single drug antiplatelet regimen with aspirin alone after TAVI, and laparoscopic colectomy was performed 22 days after TAVI with perioperative heparin bridging. We selected this treatment regimen, even though dual antiplatelet therapy using clopidogrel and aspirin is recommended for the first 6 months after TAVI, to avoid ischemic complications, because of the higher risk of tumor bleeding in our patient. Recent reports have shown that, compared with dual antiplatelet therapy, aspirin alone could reduce the risk of life-threatening/major bleeding while not increasing the risk of ischemic events following TAVI [13]. Although the preventive effects of aspirin alone in cancer patients with thrombotic tendencies remain unknown, we treated our patient with aspirin alone for fear of tumor bleeding. It is possible that heparin bridging may not have been necessary because of its unclear benefit, and one meta-analysis has shown that antiplatelet therapy at the time of non-cardiac surgery is associated with minimal bleeding risk [14]. Thus, it is possible that the colectomy could have been performed earlier in the absence of heparin bridging.

Due to factors such as age, heart failure, and cancer, 30–50% of symptomatic AS patients are considered ineligible for SAVR [15–17]. Previously, elderly cancer patients with severe AS similar to our patient may have been forced to forego treatment of severe AS and cancer, but TAVI can potentially help these patients. To the best of our knowledge, this is the first report of a patient undergoing laparoscopic colectomy for cancer after TAVI even though a few surgical reports of cancer surgery after TAVI are available [18–20]. There is no evidence on the feasibility of TAVI in severe AS patients scheduled for cancer surgery and its effects on subsequent surgery. Further studies are required to assess variables such as adequate treatment interval between TAVI and cancer surgery, a perioperative antiplatelet agent, and surgical indications.

## Conclusion

TAVI facilitated a subsequent laparoscopic colectomy in an elderly cancer patient with severe AS. Careful preoperative examination, combined with less invasive procedures, such as TAVI and laparoscopic colectomy, enabled our patient to be treated without any deterioration in general condition. TAVI may increase treatment options in cancer patients considered ineligible for certain therapeutic strategies due to severe AS.

## Data Availability

The authors declare that all data in this article are available within the article.

## Abbreviations

AS: Aortic stenosis
CPB: Cardiopulmonary bypass
SAVR: Surgical aortic valve replacement
TAVI: Transcatheter aortic valve implantation
